# Genetic Differentiation and Widespread Mitochondrial Heteroplasmy among Geographic Populations of the Gourmet Mushroom *Thelephora ganbajun* from Yunnan, China

**DOI:** 10.3390/genes13050854

**Published:** 2022-05-11

**Authors:** Haixia Li, Jianping Xu, Shaojuan Wang, Pengfei Wang, Wanqin Rao, Bin Hou, Ying Zhang

**Affiliations:** 1 State Key Laboratory for Conservation and Utilization of Bio-Resources in Yunnan, Yunnan University, Kunming 650032, China; lhx1005@mail.ynu.edu.cn (H.L.); jpxu@mcmaster.ca (J.X.); wsj20150915@163.com (S.W.); 15288453604@sina.cn (P.W.); 2 School of Life Science, Yunnan University, Kunming 650032, China; raowanqin@mail.ynu.edu.cn (W.R.); houbin@mail.ynu.edu.cn (B.H.); 3 Department of Biology, McMaster University, Hamilton, ON L8S 4K1, Canada

**Keywords:** mitochondrial inheritance, population heteroplasmy, genetic differentiation, edible mushrooms, mito-nuclear interactions

## Abstract

The mitochondrial genomes are generally considered non-recombining and homoplasmic in nature. However, our previous study provided the first evidence of extensive and stable mitochondrial heteroplasmy in natural populations of the basidiomycete fungus *Thelephora ganbajun* from Yunnan province, China. The heteroplasmy was characterized by the presence of two types of introns residing at adjacent but different sites in the cytochrome oxidase subunits I (*cox1*) gene within an individual strain. However, the frequencies of these two introns among isolates from different geographical populations and the implications for the genetic structure in natural populations have not been investigated. In this study, we analyzed DNA sequence variation at the internal transcribed spacer (ITS) regions of the nuclear ribosomal RNA gene cluster among 489 specimens from 30 geographic locations from Yunnan and compared that variation with distribution patterns of the two signature introns in the *cox1* gene that are indicative of heteroplasmy in this species. In our samples, evidence for gene flow, abundant genetic diversity, and genotypic uniqueness among geographic samples in Yunnan were revealed by ITS sequence variation. While there was insignificant positive correlation between geographic distance and genetic differentiation among the geographic samples based on ITS sequences, a moderate significant correlation was found between ITS sequence variation, geographical distance of sampling sites, and distribution patterns of the two heteroplasmic introns in the *cox1* gene. Interestingly, there was a significantly negative correlation between the copy numbers of the two co-existing introns. We discussed the implications of our results for a better understanding of the spread of stable mitochondrial heteroplasmy, mito-nuclear interactions, and conservation of this important gourmet mushroom.

## 1. Introduction

Fungi are important constituents of the global biosphere. For example, a large group of mycorrhizal fungi form symbiotic associations with over 80% of land plants [1], helping plants obtain essential minerals and water from the soil and contributing to plants’ disease resistance and stress tolerance [2]. As an important mycorrhizal fungus, *Thelephora ganbajun* not only forms symbiosis with a variety of pines and cedars, but also is known as a valuable gourmet mushroom in Yunnan Province for its unique and attractive flavor, comparable to that of dry-cured beef. During the rainy season from June to October each year, the price of this mushroom could reach as high as RMB 1500–2000 Yuan per kilogram in wild mushroom markets in Yunnan province, southwestern China [3].

Mitochondria are cell organelles that encode components of vital cellular electron transport chain and ATP synthesis complexes. All mitochondria contain their own mitochondrial DNA (mtDNA) and most eukaryotes have mitochondria in their cells. This oxidation respiratory function of mitochondria can impact the mitochondrial mutation rate and influence the genetic variation of mtDNA. Beyond ATP production, mitochondria have other cellular roles such as calcium signaling, aging, apoptosis, and nuclear genome stability [4,5]. As a result, the natural selection, adaptation, and dispersal of organisms could be reflected by the spatial distribution of mitochondrial genotypes among individuals and populations. Over the last 20 years, the rapid developments in DNA sequencing technologies have propelled unprecedented advances in biomedical research, including our understanding of the mitochondrial genes and genomes at the population level [6], shedding new light on enigmatic and important mitochondrial variation and evolutionary histories in eukaryotes [4,6,7,8,9,10,11]. Indeed, mitochondrial genome sequences have been frequently used as molecular markers for studying the population genetics of many eukaryotes, including fungi [12].

In the great majority of sexual eukaryotes, the mtDNA are inherited uniparentally with a preference for the maternal parent, without recombination or heteroplasmy [13,14,15]. However, researchers have identified a great diversity of mitochondrial inheritance patterns during sexual mating in fungi, ranging from uniparental to biparental and to the frequent generation of recombinant genotypes [5,10,16,17]. Members in the two major groups of fungi, Ascomycota and Basidiomycota, have shown very different mtDNA inheritance patterns. For instance, in the ascomycete yeasts such as *Saccharomyces* *cerevisiae* [14] and *Schizosaccharomyces* *pombe* [18], mtDNA transmission was biparental such that both mating cells contribute to the zygote mtDNA. Among American *Saccharomyces paradoxus* lineages, biparental mtDNA transmission was identified during recent hybridizations [19,20]. The basidiomycete yeast *Cryptococcus neoformans* showed uniparental mtDNA inheritance in opposite-sex mating but biparental mtDNA inheritance in same-sex matings [21]. In *Cryptococcus gattii*, both uniparental and biparental mtDNA inheritance have been found, and the inheritance patterns were often strain and strain-pair specific [22]. In many filamentous basidiomycetes, sexual mating involves unidirectional or reciprocal nuclear migrations but mitochondria do not migrate. Consequently, mated colonies contain uniform nuclear genomes but mosaics in the mitochondrial genome with some cells contain mitochondria from one parent, while others have mitochondrial genomes from a different parent. For fungi with no or limited nuclear migration, such as the commercial button mushroom *Agaricus bisporus,* heteroplasmy and mitochondrial recombination can be common in their mating products, which are typically only limited to the junction zone where two colonies meet [23,24].

Heteroplasmy is frequently associated with biparental mtDNA inheritance and mtDNA recombination [25]. However, heteroplasmy generally appears only in laboratory crosses and is typically transient. This is because vegetative growth and cell division can quickly lead to the fixation of a single mitochondrial genotype within each cell lineage [14]. However, with PCR and deep sequencing, an increasing number of studies have reported heteroplasmy in human and animal cells in their natural state [26,27,28,29]. However, the frequency of heteroplasmy and their geographic distributions in natural fungi populations are unknown. In a previous study, we provided evidence of heteroplasmy in natural populations of the basidiomycete fungus *T. ganbajun.* Stable heteroplasmy within the *cox1* gene was observed to occur at a high frequency in *T. ganbajun* samples (262/299) from 28 geographic populations in Yunnan province. In this case, heteroplasmy was manifested by the presence/absence of introns at two different but adjacent sites within the *cox1* gene [25]. Similarly, we also found evidence for heteroplasmy and mitochondrial recombination in natural populations of two other basidiomycete mushrooms, *Russula virescens* and yellow chanterelles from Yunnan [13,30]. 

Owing to the diverse climate, geology, and geography, southwestern China (especially Yunnan province) has the most abundant mushroom diversity in China, where more than 40% of the world’s and 90% of the Chinese mushroom species (about 900 species) have been found [31,32]. However, due to difficulties in artificial cultivation and aggressive picking, many of the wild edible mushrooms have shown signs of decline in local populations, including the three species with reported mitochondrial heteroplasmy, *T. ganbajun*, *R. virescens*, and yellow chanterelles [12,30]. At present, the potential genetic effects of declining mushroom populations and how mitochondrial genomes may contribute to population dynamics in these mushrooms are unknown.

In *T. ganbajun,* while evidence for mitochondrial heteroplasmy and recombination have been observed, their potential roles in the adaptations to their natural environments remain unexplored. In the human pathogenic yeast *Cryptococcus neoformans*, it has been demonstrated that high temperatures and UV exposure can change the mitochondrial inheritance pattern from uniparental to biparental [4,33], as well as promoting mitochondrial heteroplasmy and recombination. However, the observed heteroplasmy was transient in *C. neoformans* and has not been reported among natural isolates. At present, the high-frequency observation of seemingly stable heteroplasmy seems to be unique among mushroom species in Yunnan. It is tempting to speculate that such heteroplasmy may provide an advantage to these mushrooms in their local adaptations to the high UV exposure and high daily temperature fluctuations due to the high altitudes (mean altitude > 2000 m above sea level).

The combination of highly conserved and variable regions in the nuclear ribosomal DNA (the 18S, 5.8S, and 28S rRNA genes, two external transcribed spacers (ETS1 and ETS2), two internal transcribed spacers (ITS1 and ITS2), and an intergenic spacer (IGS)) of fungi allowed excellent phylogenetic inference across a broad range of evolutionary time scales to a wide range of taxa [34,35]. Most fungi possess hundreds of tandem copies of the variable ITS regions, allowed reliable amplification; the process of concerted evolution ensured that these copies were similar within individuals [36]; the functional constraints yielded evolutionary insights that morphology and coding-sequences could not provide. Recently, caution has been called for since intrastrain or intraspecies variation in ITS sequences have been reported in several fungal genera and species by either PCR amplification artifacts or ITS paralogous genes in diploid/dikaryotic organisms [37,38]. However, results have demonstrated that the genetic differences were only among nuclei for rDNA in the arbuscular mycorrhizal fungi *Glomus geosporum*, *Glomus mosseae* and *Gigaspora margarita* [39], and sequence variation in ITS regions is still widely used to indicate the evolutionary relationships in a huge number of fungal species [35] and intraspecies genetics in diploid mushroom species *Amanita citrinoannulata* [40], *Armillaria* and related species [41], and *Russula senecis* [42]. Moreover, significant genetic divergence within *T. ganbajun* and limited but detectable gene flow among geographical populations of this endemic ectomycorrhizal gourmet mushroom were detected by ITS too [1]. To further understand heteroplasmy in *T. ganbajun*, we sampled and analyzed the ITS sequence variation and *cox1* mitochondrial intron variations from 30 geographic locations in Yunnan province in southwest China. The relative copy numbers of the two types of introns in the *cox1* gene were further investigated within 176 representative samples from 18 populations. The variations in *cox1* intron distributions were discussed in the context of variations among geographic locations and ITS sequences.

## 2. Materials and Methods

### 2.1. Sampling, DNA Extraction, PCR Amplification, and Sequencing

**Figure 1:**
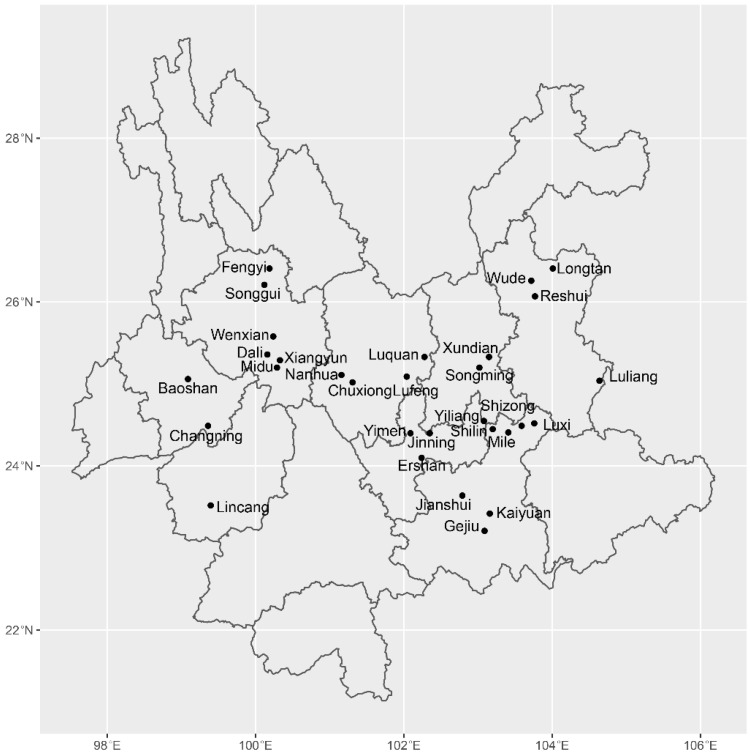
Geographical distribution of *Thelephora ganbajun* samples collected from Yunnan Province and analyzed in this study. Note: the full names of the sampling sites are indicated in Table 1.

A total of 489 fruiting bodies were obtained from 30 sites in ten municipalities of Yunnan province in southwest China. Among these 489 fruiting bodies, 221 from 23 sites were collected and analyzed for the first time, 238 samples from the 14 sites were originally reported in Wang et al.’s study [25], and the remaining 30 samples came from 4 sites as reported in the Sha et al. study [1]. Together, these 30 sites stretched about 600 km from east to west and about 350 km from south to north. The geographical locations of the sampled sites are shown in Figure 1. The geographic coordinates and the sample size from each site are presented in Table 1. DNA extraction, PCR, sequencing, and sequence alignment all followed those of Wang et al. [25]. The ITS region was amplified using primers ITS4 (5′ TCCTCCGCTTATTGATATGC 3′) and ITS5 (5′ GGAAGTAAAAGTCGTAACAAGG 3′), and the reaction conditions were as follows: a pre-denaturation step at 95 °C for 5 min, followed by 35 cycles of denaturation at 94 °C for 40 s, annealing at 52–55 °C for 50 s, and elongation at 72 °C for 60 s, followed by a final elongation at 72 °C for 10 min, after which the reaction was kept at 4 °C until gel electrophoresis. After electrophoresis, the resulting PCR product was approximately 700 bp, purified, and then was sequenced using an ABI3730XL automated DNA sequencer. Sequencing was carried out for both strands using the forward and reverse primers. In sequence chromatographs, all heterozygous sites were coded using the following universal ambiguity codes system: T/C = Y, A/G = R, A/C = M, G/T = K.

In a total of 489 mushroom fruiting bodies, the heterozygous *cox1* genes were characterized by having the two types (α and β) of introns residing at adjacent sites at variable frequencies. α and β introns were 296 bp and 312 bp, respectively. To further investigate the relative copy numbers of α/β type of introns within individual cells, 176 representative samples from 18 populations were selected. Real-time quantitative PCR tests were conducted and the nuclear single copy gene β-tubulin was used as reference. The two introns were successfully amplified in all 176 samples using the specific primer pairs described in Wang et al. [25]. We used SYBR Green System (10 µL) and the reaction conditions were as follows: a pre-denaturation step at 95 °C for 5 min, followed by 40 cycles of denaturation at 95 °C for 10 s, annealing at 60 °C for 30 s, and the dissolution curve was measured at 95 °C for 15 s, 60 °C for 60 s, 95 °C for 30 s, and 95 °C for 15 s, respectively. The 2^−ΔΔCt^ algorithm [43] was used to calculate the relative copy number of the α and β types of introns within each sample.

### 2.2. Data Analysis

#### 2.2.1. Haplotype Inference of ITS Sequences

In the case of single nucleotide polymorphisms (SNPs), each nucleotide site in each individual specimen may be homozygous for a specific nucleotide or heterozygous, containing two different nucleotides. Due to multi-copy nature of ITS sequence, heterozygotic sites were frequently found in this diploid basidiomycete, and a significant proportion (∼30%) of recombinant molecules found in ITS among the mushroom *Agaricus subrufescens* samples when divergent alleles were mixed as templates in the same reaction [38]. In our chromatographs, only nucleotide sites with double peaks of comparable peak height were scored as being heterozygous at the specific sites, and both of the two segregating alleles indicated by the double peaks were observed in all of the other samples with clean chromatographs. These two criteria eliminated the minor variants among the ITS repeat units within individual haploid nuclei but identified the variable ITS sequences between the two haploid nuclei. We inferred the putative haplotype sequences for each individual mushroom at the ITS region using the Bayesian method implemented in the program PHASE 2.1 (University of Washington, Washington, USA) [44].

#### 2.2.2. ITS Genetic Diversity and Population Structure

Haplotype diversity (Hd) was calculated for each population and each region using DnaSP v6.10.01 software (Universitat de Barcelona, Catalonia, Spain) [45]. A Nei’s pairwise genetic distance matrix among populations was generated using GenAlEx 6.5 software (The Australian National University, Canberra, Australia) [46]. The same software was used to perform the analysis of molecular variance (AMOVA) to estimate the relative contributions of geographic separation (within local populations (Phi-PT), among local populations within regions (PhiPR), and among regional populations (Phi-RT)) to the overall genetic variation. Mantel test was used to detect potential correlations between the levels of genetic differentiation, geographical distances, and heteroplasmy ratio among populations [47]. Both the Mantel test and AMOVA were conducted using GenAlEx 6.5. Furthermore, we used the PopART v1.7 (University of Otago, Otago, New Zealand) [48] to build the relationship among haplotypes inferred from PHASE 2.1 software.

Aside from the above population genetic analyses, we also estimated the number of genetic clusters using the program STRUCTURE version 2.3.3 (Stanford University, Stanford, CA, USA) [49]. The STRUCTURE program uses a Markov Chain Monte Carlo (MCMC) algorithm to estimate allele and genotype frequencies in each cluster and provide the likely population memberships for every individual. The STRUCTURE program has been used for identifying cryptic population structure, detecting migrants, and inferring historical population admixture [50,51,52]. In our analyses, a total of 30 simulations were performed for K ranging from 1 to 30 to verify the convergence of the Log likelihood values for each value of K. After the optimal K was determined, a final parameter of 1 million MCMC replicates and a length of burn-in period of 100,000 were run for the assignment of individuals into K populations. Here, our STRUCTURE output files were uploaded as a compressed file (.zip) to the site directly to obtain the optimal number of K in our samples. We used CLUMPAK (Cluster Markov Packager Across K) to process the STRUCTURE output files [53], then used DISTRUCT v1.1 (University of Southern California, Los Angeles, USA) to output structure graphics [54].

#### 2.2.3. Phylogenetic Analyses

In order to investigate the phylogenetic status of *T. ganbajun* in the genus *Thelephora*, we constructed a phylogenetic tree of eight species based on their ITS sequences. All ITS sequences obtained from our specimens and those from GenBank representing the diversity of species closely related to *T. ganbajun* were aligned by using MEGA v6.06 (Tokyo Metropolitan University, Tokyo, Japan) [55] and checked manually by BioEdit 7.1.9 (Abbott Laboratories, CA, USA) [56]. Because of the relatively large sample sizes for each local population of *T. ganbajun*, we identified and removed the duplicated ITS sequences from each local population to improve the clarity of the phylogenetic tree. The species of *Boletus edulis* (accession number EU554664) was used as outgroup. Maximum-likelihood (ML) analysis was conducted by using RAxML v7.0.4 (Ludwig-Maximilians-Universitt München, Freistaat Bayern, Germany) with the PHY files generated with CLUSTAL_X v. 1.83 (Institut de Genetique et de Biologie Moleculaire et Cellulaire, Grand Est, France), using the GTR+γ model. ML bootstrap proportions (MLBPs) were computed with 1000 replicates [57].

## 3. Results

### 3.1. ITS Haplotypes’ Distribution and Diversity

**Figure 2:**
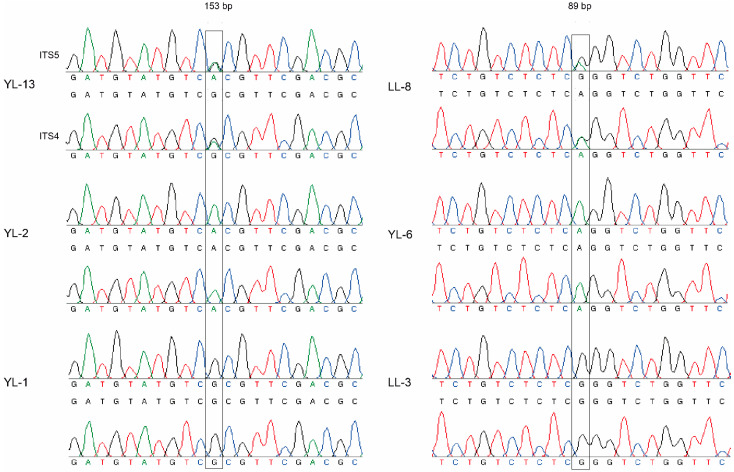
Chromatographs with clear ITS heterozygosity. Notes: Each sample had two chromatographs in different directions, the upper one was from the forward primer ITS5, and the lower chromatograph was from the reverse primer ITS4. “153 bp” and “89 bp” indicate the heterozygous position, and different colored peaks represented different nucleotide bases (black: G; green: A; blue: C; red: T).

In the study, a total of 489 specimens were collected from 30 geographic locations distributed in ten municipalities: Baoshan, Chuxiong, Dali, Honghe, Kunming, Lincang, Qujing, Weishan, Yuxi, and Xuanwei (Table 1). The ITS sequence similarities between our samples and those of *T. ganbajun* from GenBank were all over 99%. The 489 aligned sequences were 535 bp long and contained 70 variable sites. Among them, 17 sites were scored as heterozygotic point mutations and useful to indicate the variable ITS sequences between the two haploid nuclei in our samples. Figure 2 shows chromatographs with clear ITS heterozygosity and the detailed nucleotide compositions at the 17 polymorphic positions are summarized in Table S1. For example, YL-3 was from the 34 samples with heterozygous A and G on the position 153bp, and the major haploid allele G with clean chromatograms was observed at the same position in the other 446 samples represented by YL-1, and minor haploid allele A appeared in nine samples represented by YL-2. There were no sequences of ITS with unreadable chromatographs on both strands in our study; the chromatographs that were not readable on one strand were replaced by the other clean strand. Further phylogenetic analyses of our ITS sequences and the closely related ones from GenBank confirmed that all 489 samples clustered into a single clade (Figure S1), which was closest to the type strain of *T. ganbajun*.

The 489 ITS sequences were grouped into 70 haplotypes. The detailed distributions of the 70 haplotypes are shown in Table S2. Among the 70 haplotypes, 51 were found only in one sample each while the remaining 19 were shared by two or more samples each. The 51 singleton haplotypes were distributed across 19 local populations. The most common haplotype of the 19 shared haplotypes was H29, which appeared 403 times and was distributed in 29 of the 30 local populations, only the Jianshui population did not have H29. H63 was also widely distributed—it appeared a total of 180 times among 22 local populations. The other major shared haplotypes, H7 and H9, were each distributed in 18 different local populations. The H7 haplotype appeared a total of 95 times, while the H9 haplotype appeared 62 times (Table S2).

The number of ITS haplotypes varied among local populations. Specifically, the number of ITS haplotypes for each local population ranged between one in Chuxiong (H29) to 22 (Kaiyuan) (Table 1). Of these 30 local populations, 19 were found to contain private ITS haplotypes: six local populations (LF, GJ, ML, XD, LL, WD) had one private haplotype each, while the remaining 13 had two to ten private haplotypes each. The Kaiyuan population had both the largest number of total (22) and private haplotypes (10). Though the most-commonly shared haplotypes had broad geographic distributions, several commonly shared ones had limited geographic distributions (Table 1). For instance, H36 was found in DL and SG local populations, and these two locations belonged to the same region—Dali municipality. Among the 30 local populations, the ITS haplotype diversity ranged from a low of 0 in CX to a high of 0.892 in SG. Overall, the ITS haplotype diversity for the whole sample of 489 individuals was 0.781 (Table 1). 

### 3.2. Population Structure Based on ITS Sequences

**Figure 3:**
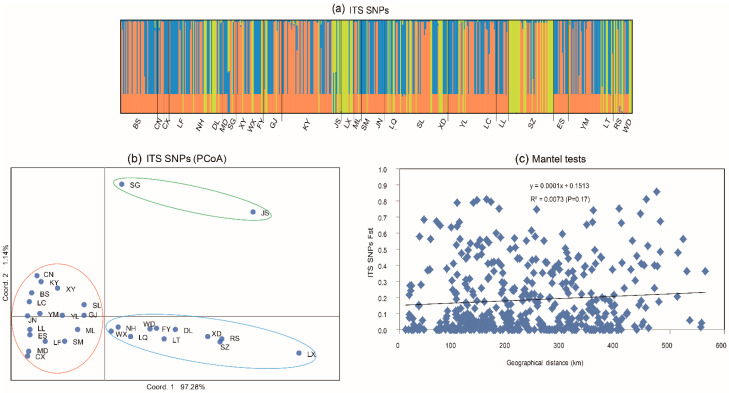
(**a**) Genetic clusters with STRUCTURE based on ITS SNPs; (**b**) principal coordinate analysis of 30 populations of *T. ganbajun* in Yunnan based on ITS Nei’s genetic distance using GenAlEx; (**c**) Mantel tests of the relationships between genetic differentiation (*Fst* values) and geographical distance indicated by ITS SNPs.

Our analyses using data from the 70 SNPs in the ITS gene fragment revealed evidence of sample clusters by their geographic origins. The number of genetic clusters K=3 was inferred using STRUCTURE analysis (Figure 3a). However, each of the three genetic clusters contained strains from most geographic populations, and all geographic populations except two (SG and JS) contained strains from two of the three genetic clusters. A similar clustering pattern was obtained by PCoA analyses (Figure 3b). The axes 1 and 2 of the PCoA accounted for 97.28% and 1.14% of the ITS genetic variation. Seventeen populations from six regions were clustered in one group in the red quadrants, while the remaining 11 populations clustered in two groups in the blue and green quadrants.

It is worth noting that there was no strong geographical isolation of the PCoA clusters for ITS. However, within certain regions, we found some small clusters of strains from geographically adjacent populations. For example, BS and CN belonging to Baoshan region, XY, WX, and FY belonging to Dali region, and LT, RS, and WD belonging to Xuanwei region were clustered based on STRUCTURE analysis of ITS sequences (Figure 3b).

### 3.3. Genetic Differentiation of ITS

A range of *Fst* values between pairs of local populations was found. The ITS *Fst* values between populations of *T. ganbajun* varied from 0 to 0.860. The lowest value (0) was found in 103 pairs out of the total of 435 ((30 × 29)/2 = 435) pairwise comparisons. The highest (*Fst* = 0.860; *p* = 0.01) was found between BS and LX, separated by about 475 km (Table S3). The pairwise comparisons of the same dataset showed that among the total 435 local population pairs, 168 pairs showed statistically significant differentiation (*p* < 0.05) based on the SNPs of the ITS gene fragment.

The analysis of molecular variance (AMOVA) of the ITS sequences revealed that 27% of the total observed genetic variations were partitioned among populations, and 73% within geographic populations (Table S4). 

Mantel tests were conducted to determine whether the observed genetic differentiation was related to geographical distances. Overall, the analyses showed an insignificant positive correlation between genetic difference and geographical distance among these 30 populations (Figure 3c).

### 3.4. Intraspecific Clustering of ITS Sequences

**Figure 4:**
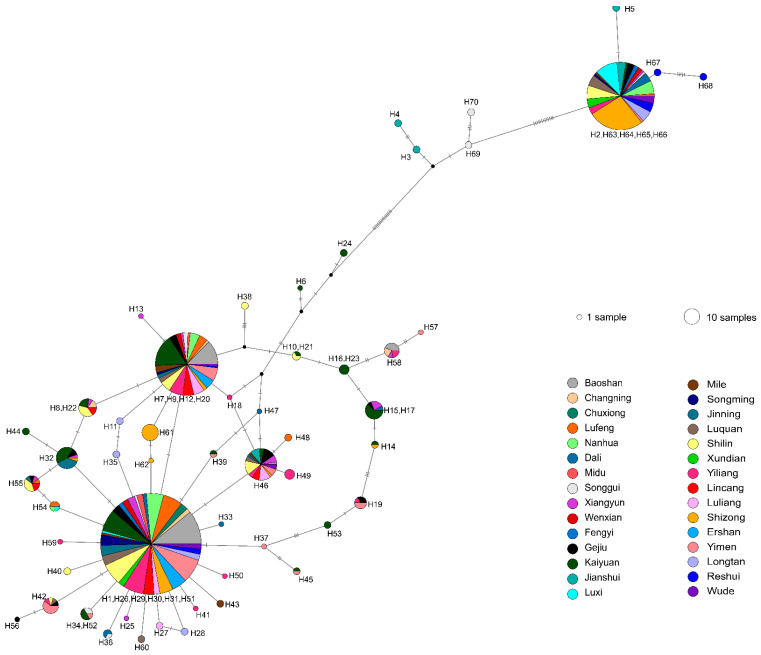
ITS haplotype networks inferred by PopART from 30 local populations of Yunnan. Notes: Each pie chart represents single or combined haplotypes. Because we used the haplotypes inferred by the PHASE software to construct the network, the PopART combined the several haplotypes with small base differences into one pie. Circle sizes indicate haplotype frequencies (number of individuals), and colors indicate the geographic populations of where the haplotypes are found. Solid lines indicate a single nucleotide substitution, and closed circles the missing haplotypes.

All ITS sequences we obtained here had more than 99% sequence identity to known *T. ganbajun* strains in the NCBI database. *T. ganbajun* is also the closest related species in the database, consistent with our morphological identifications of all newly collected samples as *T. ganbajun*. The relationships among our ITS sequences are shown Figure 4. Our phylogenetic analyses identified that our ITS sequences are grouped into three distinct clades (Clade 1–Clade 3): Clade 2 included eight haplotypes (H2, H5, H63–H68) in 22 local populations and was much more genetically distant to other clades (Figure S1). Clade 3 was the closest to the root of the phylogenetic tree among the three clades and included four haplotypes (H3, H4, H69, H70). Clade 1 contained all remaining 58 haplotypes (H1, H6–H62). By calculating the within and between clade genetic distances, including “intra“ and “inter” species distance with the known species *T. ganbajun* (Table S5), we found the distance between clade 1 and *T. ganbajun* (0.0054) was lower than the maximum intraspecies (clade 3) genetic distance value 0.018, suggesting that clade 1 is the same species as *T. ganbajun*. However, clade 2 and 3 showed much higher values than 0.018, indicating potential phylogenetic species represented by these two clades; further morphological and other molecular markers are needed to confirm. 

### 3.5. Frequencies of α and β Type of Introns in cox1 Gene

Relative to the nuclear gene β-tubulin copy number, the exact copies for both the α and β introns were calculated in representative 176 fruiting body samples from 18 geographic populations to infer their frequencies within each sample. The copy numbers of these two heterogeneous introns varies widely among samples (Table S6). The relative copy number of the α fragment in the whole 176 samples’ dataset ranged from 5.77 × 10^−^^5^ to 7488.10, and the β fragments ranged from 1.95 × 10^−^^5^ to 31850.44. On the whole, in most of the samples (138/176), the β fragment was the main mtDNA type. Among the specimens, the β type was 2.81 to 4,088,364 folds of the α type, and copy numbers of the two co-existing introns was significantly and negatively correlated (Rxy = −0.121, *p* = 0.01).

We further quantified the differences between the copy numbers of α and β introns, or their ratios, and investigated how those differences might be related to the genetic relationships inferred based on ITS sequences or to geographical distances between specimens (Table 2). Specifically, the observed differences between the individual copy numbers of α introns and α/β ratios showed significant positive correlation with both the ITS sequence difference and their geographical distances. In contrast, those of the β intron showed the reverse pattern and that the correlation between the individual copy number differences and geographical distances was not significant.

## 4. Discussion

### 4.1. Distinct Intraspecific Differentiation Based on ITS Sequences

The ITS sequence is typically present in multiple copies in each haploid fungal genome. The fruiting bodies of *T. ganbajun* are heterokaryotic, containing two different nuclei within each cell. In the case of multi-copy ITS sequences, heterozygotic sites can be generated by multi-template PCR containing different ITS paralogous genes, which can be slightly different in nucleotide sequences. If the ITS paralogous genes were co-amplified, then it would be problematic to infer the putative haplotype sequences for each individual mushroom. Thus, taking into account the various complications, we tried to score the heterozygous sites in ITS sequences observed in our specimens firstly. Consequently, the polymorphic ITS haplotypes identified here could be used to infer nuclear gene flow and population structure. 

In this study, we analyzed 489 strains of the endemic gourmet mushroom from 30 geographic populations in Yunnan. The results identified abundant ITS sequence types, while the phylogenetic results suggested that the analyzed samples all belong to the same species *T. ganbajun*. According to the BI tree topology, our ITS sequences were grouped into three distinct clades, and the haplotype distribution of each local population among the distinct clades was supported by the strong divergent signals in the haplotype network analyses (Figure 4 and Figure S1). High levels of genetic variations detected from a relatively small geographic area suggest that this mushroom represents an excellent system from which to examine the population biology and genetic variations of mushrooms in nature. 

### 4.2. Genetic Variation and Differentiation Revealed by ITS Sequences

Our study characterized the ITS genetic variation of *T. ganbajun* isolated from Yunnan province. The ITS results showed that there was a high genetic diversity and genetic differentiation among geographic populations. However, evidence for gene flow was also found. Dry windy climates have been assumed to contribute to the long-distance spore dispersals [1,30,47]. A similar process could have accounted for the wide distribution of ITS haplotype 29 in our samples. The existence of gene flow was further supported by results from STRUCTURE analysis that showed that there were three widely dispersed genetic clusters (K = 3), and most geographic populations contained ITS from all of the three genetic clusters. In addition, there was no correlation between genetic distance and geographic distance among specimens by Mantel test. On the other hand, although the overall genetic variation in the total samples was not geographically correlated, specimens within several local geographical populations (e.g., sites within Baoshan, Dali, and Xuanwei) were tightly clustered within each other. Together, our analyses suggested that the population structure of *T. ganbajun* in Yunnan is likely influenced by multiple factors, including historical divergence, recent gene flow, and geographic distances within several municipalities. Geographic separation has been shown to be an important contributor to genetic differentiation in many species [58]. Baoshan and Dali are close to each other in the northwest of Yunnan province, while Xuanwei is located in the northeast part of Yunnan, the straight-line distance between them is about 500 km, representing the farthest distance between pairs of geographical populations we selected in this study. Interestingly, these two pairs of populations also showed among the highest level of genetic differentiation, consistent with their geographic separation and limited long-distance gene flow. However, environmental changes due to global warming as well as anthropogenic factors can facilitate gene flow and/or local adaptation and differentiation [59]. 

Our results showed higher genetic diversity and significant genetic differentiation than previous results. A population study based on ITS sequences to identify the genetic diversity of *T. ganbajun* from Yunnan province was published 13 years ago. Sha et al. investigated 156 specimens collected from 23 sites in nine regions in Yunnan province. Though both studies showed that most of the observed ITS sequence variation was within local populations, our study’s overall and pairwise *Fst* values were higher than those of Sha et al. Our analysis of AMOVA revealed that 27% of the total observed genetic variations were among populations, and those of Sha’s study were 7.4%. The following possible factors could have contributed to the increased genetic variation found among populations: (i) the increased sample size in the current study; (ii) the broader regions included in this analysis; (iii) increasing application of artificial propagation and conservation of this species in their native forests during the last decade, which potentially could have facilitated more fruiting opportunities of local germplasm [60].

### 4.3. Distribution of Heteroplasmic Mitochondrial cox1 Gene

Quantitative real-time PCR and estimation of the frequency and distribution pattern of the two types of mtDNA represented by the intron types α and β within the *cox1* gene were performed on 176 specimens from 18 geographic populations. All 176 specimens contained both mtDNA types. However, the frequencies of these two mtDNA types among the specimens differed. In most specimens, the β intron type was more common than the α intron type. The moderate but statistically significant correlations between the individual copy numbers of the α and β mtDNA types as well as their ratios with ITS sequence divergence suggest that their copy numbers and ratios are likely evolutionarily stable and of adaptive significance.

Heteroplasmy could contribute to increased population genetic diversity, as a buffer against population bottlenecks, and to help maintain population stability [61]. In the basidiomycetous species *Phellinus noxius*, heteroplasmy was associated with mitochondrial exchange during mating and hyphal fusion, resulting in recombinant mitochondrial genotypes [62]. Transmissions of heteroplasmy have been observed in fungi and several animals, and these were explained by selection pressure [63] or the stringent replication and partition of mitochondrial nucleoids [13,26,64]. The potential selection factors for *T. ganbajun* include significant UV exposure and high temperature, both of which could favor mitochondrial heteroplasmy and recombination as an adaptive response to reduce the rate of accumulation of deleterious mutations in the mitochondrial genome [4,33]. Heteroplasmy may be generated from mating or inherited from a heteroplasmic parent (usually the maternal parent in animals) [20,63,65]. Here, due to the apparent stability of heteroplasmy among specimens, we hypothesize that the existence of heterogeneity in natural populations is likely caused by the inheritance of mitochondria from heteroplasmic parental cells and that such stable and broadly distributed heteroplasmy in *T. ganbajun* in Yunnan likely represented an adaptive response to the local environmental conditions. 

At present, the mechanism(s) for the observed correlation between the nuclear ITS sequence divergence and mitochondrial heteroplasmy in *T. ganbajun* is not known. However, there have been examples of significant nuclear–mitochondrial interactions affecting strain fitness. For example, in the yeast *Saccharomyces cerevisiae*, over time, a mixture of mtDNA molecules from two different strains slowly “drifted” into a situation where the original mtDNA outcompeted the donor mtDNA to reconstitute the original mtDNA-nDNA genotype combination [66,67]. In addition, the mitochondrial genome copy number in *S. cerevisiae* is related to the nuclear genome and linearly scales up with ploidy [68]. A study on human cell lines has also shown that the nuclear background can predictively select the mtDNA haplotype that they will coexist with, largely based on their prior associations [69]. This suggests that the dynamics of heteroplasmy could be non-random but might have coevolution with its nuclear background [70]. Taken together, genetic compatibility between the mitochondrial and nuclear genomes might shape heteroplasmy dynamics in individuals and in populations. The observed heteroplasmy in *T. ganbajun* could potentially serve as a model for a better understanding of mitochondrial–nuclear genome interactions and the heteroplasmy inheritance mechanism.

In conclusion, this study revealed extensive ITS sequence variation and *cox1* heteroplasmy within and among populations of the gourmet ectomycorrhizal mushroom *T. ganbajun*. Our study identified evidence for gene flow and significant genetic differentiation among local and regional populations of this species in its endemic range in southwestern China. These *T. ganbajun* populations with abundant genetic variation and diverse mitochondrial heteroplasmic patterns are excellent materials for our future study of mitochondrial inheritance and adaptive evolution. The periodic monitoring of the population genetics of the species, with a focus on the species-specific characteristics, including its life history and habitat, could help its conservation and utilization. Due to the multi-copy nature of ITS sequences, the co-amplified ITS paralogous genes could lead to various complications to infer nuclear genetic diversity in our samples. Therefore, it is necessary to conduct an in-depth comparative analysis at the mitochondrial genome level and single nuclear genes to find the interactions between hot spots of mitochondrial DNA heteroplasmy and nuclear DNA variance in the future.

## Figures and Tables

**Table 1:** Distribution and diversity of ITS sequence types for *Thelephora ganbajun* from Yunnan province, southwestern China.

Region	Geographic Population	Long. (E)	Lat. (N)	Sample Size	Number of Haplotypes (No. of Private Haplotypes)	ITS Haplotype Diversity
Baoshan	Baoshan (BS)	99.09	25.06	35	7 (2)	0.509
	Changning (CN)	99.36	24.49	6	4	0.727
Chuxiong	Chuxiong (CX)	101.31	25.02	5	1	0
	Lufeng (LF)	102.04	25.09	20	7 (1)	0.553
	Nanhua (NH)	101.16	25.11	22	5	0.633
Dali	Dali (DL)	100.16	25.36	9	6 (2)	0.745
	Midu (MD)	100.29	25.2	5	2	0.356
	Songgui (SG)	100.12	26.21	8	7 (2)	0.892
	Xiangyun (XY)	100.33	25.29	13	11 (2)	0.837
	Wenxian (WX)	100.24	25.58	8	4	0.667
	Fengyi (FY)	100.19	26.41	5	2	0.533
Honghe	Gejiu (GJ)	103.09	23.21	18	11 (1)	0.846
	Kaiyuan (KY)	103.16	23.42	48	22 (10)	0.824
	Jianshui (JS)	102.79	23.64	9	5 (3)	0.758
	Luxi (LX)	103.76	24.52	12	3	0.301
	Mile (ML)	103.41	24.41	7	6 (1)	0.780
Kunming	Songming(SM)	103.02	25.2	9	3	0.392
	Jinning (JN)	102.35	24.4	13	5	0.646
	Luquan (LQ)	102.28	25.33	16	8 (3)	0.736
	Shilin (SL)	103.2	24.45	36	11 (3)	0.788
	Xundian (XD)	103.15	25.33	9	3 (1)	0.523
	Yiliang (YL)	103.08	24.55	31	15 (6)	0.723
Lincang	Lincang (LC)	99.397	23.52	15	6	0.697
Qujing	Luliang (LL)	104.64	25.04	12	5 (1)	0.728
	Shizong (SZ)	103.59	24.49	43	11 (4)	0.607
Weishan	Ershan (ES)	102.24	24.1	14	3	0.423
Yuxi	Yimen (YM)	102.09	24.4	31	12 (2)	0.697
Xuanwei	Longtan (LT)	104.01	26.41	12	6 (4)	0.725
	Reshui (RS)	103.77	26.07	10	5 (2)	0.732
	Wude (WD)	103.72	26.26	8	5 (1)	0.733
Total	30			489	70 (51)	0.781

**Table 2:** Results of Mantel tests between differences of individual copy numbers of α, β introns, or their ratio, and ITS sequence difference or specimens’ geographical distances.

	α	β	α/β
ITS sequence difference	−0.101 (*p* = 0.02)	0.084 (*p* = 0.02)	−0.111 (*p* = 0.01)
Geographical distance	−0.067 (*p* = 0.06)	0.03 (*p* = 0.11)	−0.082 (*p* = 0.02)

## Data Availability

Representative ITS sequences for all the haplotypes have been submitted to GenBank; the detailed information on the accession numbers was given in supplementary Table S2.

## Supplementary Materials

The following supporting information can be downloaded at: https://www.mdpi.com/article/10.3390/genes13050854/s1, Figure S1: The maximum-likelihood tree showing the relationships among 249 representative ITS sequences of the genus *Thelephora*; Table S1: Nucleotide composition at 17 polymorphic positions in ITS sequences of Yunnan samples of *T. ganbajun*. Table S2: The distributions of ITS haplotypes of *T. ganbajun* at 30 local populations in Yunnan province; Table S3: The ITS *Fst* value of *T. ganbajun* from 30 geographic populations in Yunnan. The lower left part of the table is the *Fst* value between two populations, and the upper right part of the table is the corresponding p-value; Table S4: Results of the analysis of molecular variance (AMOVA) for ITS sequence of the *T. ganbajun* from Yunnan, southwestern China; Table S5: Average evolutionary divergence over ITS sequence pairs within and between clades (provincially adopted phylogenetic species) calculated by MEGA 6 using the K2P model. Table S6: Relative copy numbers of the α and β introns in representative 176 samples estimated by quantitative real-time PCR.
