# Supplementary material for: Genetic Differentiation and Widespread Mitochondrial Heteroplasmy among Geographic Populations of the Gourmet Mushroom Thelephora ganbajun from Yunnan, China
Source: Genes (Basel). 2022 May 11;13(5):854. doi: 10.3390/genes13050854 (PMC9141859; doi:10.3390/genes13050854)
Supplement: Supplementary file 1 [file genes-13-00854-s001.zip › Table S2 The distributions of ITS haplotypes of T. ganbajun at 30 local sites in Yunnan province..pdf]

Table S2. The distributions of ITS haplotypes of *T. ganbajun* at 30 local sites in Yunnan province.

	BS	CN	CX	LF	NH	DL	MD	SG	XY	WX	FY	GJ	KY	JS	LX	ML	SM	JN	LQ	SL	XD	YL	LC	LL	SZ	ES	YM	LT	RS	WD	Total	Accession Numbers
H1																														2	2	OM004281
H2																			2												2	OM004175
H3														2																	2	OM004132
H4														2																	2	OM004130
H5														2																	2	OM004137
H6													1																		1	KY245127
H7	17	2		4	7			4	2	2		3	12			2		3		8		6	4	3	1	8	7				95	KY245064
H8		2							1				1							5			2								11	KY245297
H9	2			3	1		2			2		3	14			4	2		2	1		6	5	6	2		4		1	2	62	OM004095
H10													1																		1	KY245145
H11																												2			2	OM004270
H12	1																														1	KY245074
H13									1																						1	KY245246
H14													1												1						2	KY245211
H15													2																		2	KY245141
H16													3																		3	OM004119
H17						1			3			1	6																		11	KY245108
H18																						1									1	OM004209
H19												2										1					3				6	KY245281
H20																			2												2	OM004177
H21																				2											2	OM004195
H22													2																		2	KY245148
H23													1																		1	KY245128
H24													2																		2	OM004121
H25									1																						1	KY245246
H26																						1				2					3	OM004253

H27																								2							2	OM004229
H28																												2			2	OM004268
H29	40	6	10	26	23	5	8	3	10	8	6	11	29		3	3	14	14	13	29	8	27	14	8	16	18	32	8	7	4	403	KY245059
H30																									2						2	KY245224
H31																						2									2	OM004211
H32									1			2	8					7							1						19	KY245113
H33						1																									1	KY245091
H34								2					2														1				5	KY245181
H35																												2			2	OM004260
H36						2		1																							3	KY245090
H37																											1				1	KY245280
H38																				2											2	OM004183
H39													1														1				2	KY245295
H40																				2											2	OM004186
H41																						1									1	KY245258
H42												1							1	1		1					6				10	KY245279
H43																2															2	OM004155
H44													2																		2	KY245156
H45													1														1				2	KY245138
H46	1								3			5	2	4		1		1	2	6		2	3	5	1		3			2	41	KY245153
H47						1																									1	KY245086
H48				2																											2	OM004081
H49																						4									4	OM004212
H50																						1									1	OM004217
H51	5																														5	KY245062
H52													1																		1	KY245128
H53													2																		2	KY245148
H54				2	1										1																4	OM004093
H55				1					1			1						1		4			2								10	OM004088
H56												1																			1	KY245093

H57																										1				1	KY245272
H58	4	2							1													2									9	OM004069
H59																						1									1	KY245256
H60																			2												2	OM004176
H61																									11						11	KY245236
H62																									1						1	KY245223
H63				2	12	8		2	2	4	4	6	2	8	20	2	2		8	12	8	6			48		2	8	8	6	180	OM004090
H64																					2										2	OM004204
H65																												2			2	OM004261
H66																									2						2	OM004239
H67																													2		2	OM004279
H68																													2		2	OM004277
H69								2																							2	KY245111
H70								2																							2	KY245110
