# Supplementary material for: Genetic Differentiation and Widespread Mitochondrial Heteroplasmy among Geographic Populations of the Gourmet Mushroom Thelephora ganbajun from Yunnan, China
Source: Genes (Basel). 2022 May 11;13(5):854. doi: 10.3390/genes13050854 (PMC9141859; doi:10.3390/genes13050854)
Supplement: Supplementary file 1 [file genes-13-00854-s001.zip › Table S3 The ITS Fst value of T. ganbajun from 30 geographic populations in Yunnan.pdf]

Table S3. Genetic differentiation between pairs of *T. ganbajun* geographic populations from 30 locations in Yunnan. The F_{ST} values between populations are shown in the lower left part of the table, and the corresponding p values are shown in the upper right part.

	**BS**	**CN**	**CX**	**LF**	**NH**	**DL**	**MD**	**SG**	**XY**	**WX**	**FY**	**GJ**	**KY**	**JS**	**LX**	**ML**	**SM**	**JN**	**LQ**	**SL**	**XD**	**YL**	**LC**	**LL**	**SZ**	**ES**	**YM**	**LT**	**RS**	**WD**
BS		0.301	0.131	0.045	0.004	0.001	0.302	0.001	0.024	0.022	0.015	0.014	0.003	0.001	0.001	0.073	0.073	0.017	0.001	0.021	0.001	0.052	0.172	0.107	0.001	0.119	0.073	0.001	0.001	0.003
CN	0.003		0.073	0.230	0.279	0.069	0.205	0.164	0.402	0.381	0.150	0.436	0.264	0.008	0.002	0.429	0.445	0.058	0.261	0.526	0.078	0.115	0.385	0.161	0.006	0.150	0.223	0.103	0.029	0.167
CX	0.088	0.161		0.194	0.065	0.107	0.001	0.040	0.290	0.233	0.464	0.142	0.169	0.015	0.001	0.455	0.355	0.114	0.146	0.182	0.094	0.177	0.059	0.195	0.001	0.255	0.189	0.183	0.063	0.106
LF	0.035	0.000	0.000		0.046	0.005	0.200	0.030	0.459	0.076	0.097	0.120	0.095	0.001	0.001	0.132	0.227	0.384	0.038	0.085	0.007	0.290	0.225	0.430	0.001	0.517	0.505	0.018	0.002	0.036
NH	0.249	0.074	0.096	0.118		0.219	0.111	0.385	0.166	0.383	0.290	0.264	0.003	0.009	0.004	0.304	0.166	0.016	0.335	0.346	0.187	0.118	0.046	0.033	0.014	0.051	0.003	0.271	0.081	0.347
DL	0.566	0.262	0.286	0.356	0.000		0.114	0.343	0.079	0.297	0.309	0.081	0.002	0.327	0.149	0.156	0.049	0.002	0.390	0.038	0.349	0.002	0.002	0.002	0.270	0.001	0.001	0.270	0.383	0.303
MD	0.017	0.062	0.000	0.000	0.085	0.277		0.063	0.306	0.256	0.471	0.217	0.278	0.018	0.005	0.451	0.348	0.171	0.262	0.229	0.085	0.194	0.190	0.385	0.001	0.375	0.171	0.199	0.076	0.176
SG	0.411	0.110	0.141	0.216	0.000	0.000	0.132		0.196	0.360	0.457	0.193	0.008	0.058	0.011	0.371	0.209	0.001	0.366	0.243	0.212	0.039	0.009	0.006	0.045	0.004	0.019	0.332	0.169	0.173
XY	0.065	0.000	0.000	0.000	0.048	0.220	0.000	0.095		0.381	0.130	0.307	0.296	0.003	0.001	0.416	0.512	0.045	0.128	0.290	0.028	0.341	0.121	0.121	0.001	0.014	0.216	0.052	0.021	0.206
WX	0.318	0.049	0.080	0.100	0.000	0.000	0.062	0.000	0.010		0.230	0.293	0.039	0.048	0.018	0.185	0.260	0.007	0.361	0.285	0.353	0.476	0.031	0.061	0.062	0.052	0.030	0.335	0.173	0.271
FY	0.578	0.218	0.250	0.310	0.000	0.000	0.237	0.000	0.155	0.000		0.504	0.018	0.337	0.121	0.269	0.532	0.041	0.292	0.247	0.261	0.133	0.026	0.053	0.390	0.057	0.040	0.376	0.287	0.565
GJ	0.140	0.000	0.010	0.027	0.000	0.104	0.000	0.029	0.000	0.000	0.015		0.050	0.005	0.001	0.473	0.261	0.069	0.286	0.216	0.064	0.369	0.167	0.136	0.002	0.103	0.081	0.131	0.021	0.290
KY	0.051	0.000	0.032	0.039	0.198	0.456	0.000	0.306	0.000	0.190	0.414	0.072		0.001	0.001	0.136	0.058	0.050	0.001	0.015	0.002	0.039	0.102	0.123	0.001	0.038	0.018	0.001	0.001	0.007
JS	0.754	0.495	0.513	0.600	0.255	0.032	0.507	0.152	0.480	0.225	0.029	0.378	0.685		0.281	0.021	0.008	0.001	0.067	0.002	0.212	0.001	0.001	0.001	0.262	0.001	0.001	0.169	0.310	0.153
LX	0.860	0.721	0.747	0.755	0.425	0.200	0.742	0.418	0.671	0.452	0.258	0.560	0.791	0.023		0.004	0.001	0.001	0.007	0.001	0.240	0.001	0.001	0.001	0.166	0.001	0.001	0.052	0.195	0.033
ML	0.157	0.000	0.000	0.000	0.000	0.076	0.000	0.000	0.000	0.000	0.000	0.000	0.047	0.345	0.581		0.452	0.007	0.307	0.377	0.132	0.332	0.057	0.230	0.016	0.108	0.049	0.288	0.124	0.329
SM	0.105	0.000	0.000	0.000	0.002	0.158	0.000	0.051	0.000	0.000	0.072	0.000	0.043	0.424	0.643	0.000		0.041	0.289	0.355	0.143	0.560	0.026	0.150	0.003	0.160	0.138	0.097	0.036	0.091
JN	0.109	0.105	0.100	0.010	0.183	0.434	0.068	0.289	0.047	0.223	0.445	0.089	0.055	0.641	0.808	0.125	0.059		0.012	0.046	0.001	0.098	0.178	0.022	0.001	0.037	0.172	0.002	0.001	0.006
LQ	0.321	0.105	0.117	0.156	0.000	0.000	0.107	0.000	0.075	0.000	0.000	0.000	0.251	0.186	0.366	0.000	0.019	0.225		0.195	0.390	0.084	0.026	0.045	0.045	0.044	0.006	0.339	0.176	0.367
SL	0.108	0.000	0.017	0.032	0.000	0.132	0.005	0.046	0.000	0.000	0.039	0.000	0.077	0.413	0.563	0.000	0.000	0.072	0.017		0.012	0.268	0.128	0.064	0.002	0.056	0.057	0.060	0.008	0.232
XD	0.666	0.377	0.401	0.475	0.084	0.000	0.392	0.071	0.343	0.057	0.000	0.219	0.571	0.000	0.083	0.191	0.277	0.541	0.026	0.248		0.009	0.002	0.009	0.273	0.002	0.003	0.373	0.351	0.327
YL	0.048	0.000	0.000	0.000	0.056	0.257	0.000	0.128	0.000	0.013	0.176	0.000	0.035	0.531	0.678	0.000	0.000	0.043	0.090	0.000	0.382		0.119	0.272	0.001	0.184	0.227	0.019	0.005	0.075
LC	0.024	0.011	0.169	0.013	0.180	0.446	0.064	0.296	0.024	0.222	0.458	0.080	0.028	0.654	0.812	0.095	0.070	0.042	0.230	0.065	0.554	0.028		0.263	0.001	0.083	0.350	0.004	0.001	0.012
LL	0.045	0.061	0.078	0.000	0.162	0.403	0.000	0.262	0.026	0.179	0.404	0.064	0.038	0.618	0.793	0.049	0.027	0.118	0.198	0.064	0.513	0.013	0.019		0.001	0.197	0.312	0.025	0.002	0.051
SZ	0.524	0.377	0.401	0.419	0.149	0.000	0.394	0.153	0.344	0.136	0.000	0.259	0.495	0.018	0.075	0.246	0.306	0.457	0.103	0.281	0.000	0.369	0.458	0.442		0.001	0.001	0.178	0.308	0.256
ES	0.035	0.074	0.136	0.000	0.174	0.438	0.030	0.293	0.062	0.208	0.450	0.090	0.051	0.648	0.811	0.084	0.048	0.152	0.220	0.076	0.546	0.030	0.081	0.041	0.455		0.201	0.006	0.001	0.017
YM	0.017	0.000	0.000	0.000	0.173	0.444	0.000	0.291	0.002	0.182	0.413	0.057	0.034	0.672	0.797	0.042	0.001	0.035	0.227	0.064	0.559	0.005	0.008	0.005	0.470	0.024		0.003	0.001	0.012
LT	0.483	0.210	0.228	0.289	0.000	0.000	0.219	0.000	0.179	0.000	0.000	0.077	0.397	0.076	0.242	0.044	0.115	0.357	0.000	0.102	0.000	0.208	0.368	0.328	0.013	0.357	0.375		0.208	0.369
RS	0.676	0.398	0.420	0.501	0.134	0.000	0.412	0.112	0.376	0.106	0.000	0.264	0.598	0.000	0.046	0.231	0.315	0.552	0.075	0.296	0.000	0.422	0.564	0.526	0.000	0.557	0.581	0.000		0.143
WD	0.489	0.177	0.200	0.259	0.000	0.000	0.188	0.000	0.133	0.000	0.000	0.021	0.365	0.086	0.293	0.000	0.064	0.357	0.000	0.046	0.000	0.155	0.366	0.319	0.021	0.358	0.352	0.000	0.000	
