# Supplementary material for: Genetic Differentiation and Widespread Mitochondrial Heteroplasmy among Geographic Populations of the Gourmet Mushroom Thelephora ganbajun from Yunnan, China
Source: Genes (Basel). 2022 May 11;13(5):854. doi: 10.3390/genes13050854 (PMC9141859; doi:10.3390/genes13050854)
Supplement: Supplementary file 1 [file genes-13-00854-s001.zip › Table S4 Results of the analysis of molecular variance (AMOVA) for ITS sequence of the T. ganbajun from Yunnan, southwestern China..pdf]

Table S4. Results of the analysis of molecular variance (AMOVA) based on ITS sequences of 489 *T. ganbajun* specimens from 30 location populations in nine municipalities from Yunnan, southwestern China.

	**Source**	**df**	**SS**	**MS**	**Est. Var.**	**%**	**Stat**	**Value**	***p*(rand >= data)**
ITS SNPs	Among Regions	9	656.338	72.926	0	0%	PhiRT	-0.017	0.904
	Among Pops	20	1077.678	53.884	3.356	27%	PhiPR	0.272	0.001
	Within Pops	459	4115.090	8.965	8.965	73%	PhiPT	0.260	0.001
	Total	488	5849.106		12.322	100%			
