# Supplementary material for: Genetic Differentiation and Widespread Mitochondrial Heteroplasmy among Geographic Populations of the Gourmet Mushroom Thelephora ganbajun from Yunnan, China
Source: Genes (Basel). 2022 May 11;13(5):854. doi: 10.3390/genes13050854 (PMC9141859; doi:10.3390/genes13050854)
Supplement: Supplementary file 1 [file genes-13-00854-s001.zip › Table S6 Relative copy numbers of the a┴ and a┬ introns in representative 176 samples estimated by Quan-titative real-time PCR.pdf]

Table S6. Relative copy numbers of the α and β mitochondrial types in 176 representative specimens. The copy numbers were estimated by Quantitative real-time PCR, using the single-copy nuclear gene beta-tubulin as reference.

Sample	Geographic population	Ct value of TUB	Ct value of α	Ct value of β	Relative copy number of α/TUB	Relative copy number of β/TUB	β/α ratio	α/β ratio
BS2-1	BS	23.88	30.21	16.27	2.48E-02	3.90E+02	1.57E+04	6.36E-05
BS2-13	BS	23.49	28.51	14.10	6.18E-02	1.35E+03	2.18E+04	4.58E-05
BS2-15	BS	24.12	30.83	15.94	1.92E-02	5.81E+02	3.02E+04	3.31E-05
BS2-16	BS	24.05	28.47	15.41	9.34E-02	7.99E+02	8.55E+03	1.17E-04
BS2-17	BS	26.24	32.31	23.22	2.98E-02	1.62E+01	5.45E+02	1.84E-03
BS2-18	BS	26.99	29.95	16.03	2.57E-01	3.98E+03	1.55E+04	6.46E-05
BS2-19	BS	25.18	30.51	15.62	4.98E-02	1.50E+03	3.02E+04	3.31E-05
BS2-2	BS	23.68	28.92	14.41	5.30E-02	1.24E+03	2.34E+04	4.28E-05
BS2-20	BS	25.21	32.04	17.07	1.75E-02	5.63E+02	3.22E+04	3.11E-05
BS2-21	BS	24.09	28.73	13.84	7.98E-02	2.42E+03	3.03E+04	3.30E-05
BS2-22	BS	24.04	26.61	12.47	3.38E-01	6.12E+03	1.81E+04	5.52E-05
BS2-24	BS	26.10	32.43	17.62	2.50E-02	7.14E+02	2.85E+04	3.50E-05
BS2-25	BS	26.17	31.19	16.31	6.13E-02	1.86E+03	3.03E+04	3.30E-05
BS2-26	BS	24.25	32.22	15.97	7.97E-03	6.20E+02	7.77E+04	1.29E-05
BS2-3	BS	26.15	34.37	18.56	6.71E-03	3.84E+02	5.73E+04	1.75E-05
BS2-5	BS	25.10	31.55	16.12	2.28E-02	1.00E+03	4.40E+04	2.27E-05
BS2-6	BS	25.36	30.03	14.47	7.86E-02	3.79E+03	4.82E+04	2.07E-05
BS2-7	BS	27.11	33.36	17.47	2.62E-02	1.59E+03	6.09E+04	1.64E-05
LF-1	CX	28.62	31.44	22.98	2.84E-01	9.94E+01	3.50E+02	2.86E-03
LF-10	CX	23.04	30.57	16.47	1.08E-02	1.90E+02	1.76E+04	5.68E-05
LF-11	CX	25.85	29.26	15.40	1.88E-01	2.80E+03	1.49E+04	6.71E-05
LF-12	CX	26.86	28.11	15.64	8.42E-01	4.76E+03	5.65E+03	1.77E-04
LF-13	CX	22.68	28.38	15.13	3.84E-02	3.73E+02	9.73E+03	1.03E-04

LF-14	CX	28.29	29.90	17.11	6.55E-01	4.63E+03	7.07E+03	1.42E-04
LF-2	CX	28.04	33.14	19.02	5.80E-02	1.04E+03	1.79E+04	5.59E-05
LF-3	CX	29.02	33.95	22.67	6.53E-02	1.62E+02	2.48E+03	4.03E-04
LF-4	CX	28.23	26.51	17.78	6.60E+00	2.81E+03	4.26E+02	2.35E-03
LF-5	CX	31.31	20.27	27.50	4.20E+03	2.80E+01	6.67E-03	1.50E+02
LF-6	CX	28.04	31.69	17.62	1.60E-01	2.75E+03	1.72E+04	5.82E-05
LF-7	CX	25.95	19.02	27.54	2.44E+02	6.64E-01	2.72E-03	3.68E+02
LF-8	CX	27.53	29.53	15.90	5.00E-01	6.35E+03	1.27E+04	7.88E-05
LF-9	CX	22.61	29.85	15.65	1.32E-02	2.49E+02	1.88E+04	5.32E-05
NH-2	CX	27.29	18.92	27.50	6.61E+02	1.73E+00	2.62E-03	3.82E+02
NH-4	CX	26.28	29.40	15.05	2.31E-01	4.83E+03	2.09E+04	4.78E-05
XY18-9 *	DL	22.54±0.16	30.69±0.07	18.11±0.2	0.058±0.01	(1.98±0.29)×10-4	3.41E-03	2.93E+02
GJ1-2	HH	28.81	20.86	35.00	4.92E+02	2.72E-02	5.54E-05	1.81E+04
JS-11	HH	24.23	28.48	16.82	1.05E-01	3.39E+02	3.23E+03	3.10E-04
JS-12	HH	26.22	19.34	25.28	2.37E+02	3.84E+00	1.62E-02	6.16E+01
JS-13	HH	24.33	31.30	18.82	1.59E-02	9.10E+01	5.72E+03	1.75E-04
JS-14	HH	32.27	36.58	24.12	1.01E-01	5.67E+02	5.64E+03	1.77E-04
JS-3	HH	24.56	12.69	28.33	7.49E+03	1.46E-01	1.95E-05	5.13E+04
JS-5	HH	22.76	27.89	16.28	5.73E-02	1.79E+02	3.13E+03	3.20E-04
JS-7	HH	26.35	28.06	14.55	6.12E-01	7.14E+03	1.17E+04	8.57E-05
JS-8	HH	24.93	28.18	16.57	2.10E-01	6.56E+02	3.12E+03	3.20E-04
KY-1	HH	21.59	22.21	26.44	1.30E+00	6.93E-02	5.33E-02	1.88E+01
KY-10	HH	29.99	19.04	28.98	3.95E+03	4.02E+00	1.02E-03	9.83E+02
KY-2	HH	23.98	39.06	17.09	5.77E-05	2.36E+02	4.09E+06	2.45E-07
KY-3	HH	29.80	29.91	18.65	1.86E+00	4.55E+03	2.45E+03	4.09E-04
KY-4	HH	29.19	20.12	30.38	1.07E+03	8.78E-01	8.17E-04	1.22E+03
KY-5	HH	28.05	28.41	18.08	1.56E+00	2.00E+03	1.29E+03	7.77E-04
KY-6	HH	29.09	31.22	29.73	4.58E-01	1.29E+00	2.81E+00	3.56E-01
KY-7	HH	27.16	23.00	33.02	3.58E+01	3.46E-02	9.65E-04	1.04E+03
KY7-11 *	HH	15.8±0.36	25.98±0.66	16.55±0.19	2.15±0.59	(1.95±0.85)×10-3	9.07E-04	1.10E+03

KY7-21 *	HH	28.13±0.37	13.28±0.17	16.16±0.18	(3.12±0.92)×10-4	8.97±1.54	2.88E+04	3.48E-05
KY-8	HH	27.45	28.13	18.17	1.25E+00	1.25E+03	9.96E+02	1.00E-03
KY-9	HH	29.51	28.00	26.40	5.70E+00	1.73E+01	3.04E+00	3.29E-01
LX-1	HH	22.81	29.85	15.71	1.52E-02	2.74E+02	1.81E+04	5.53E-05
LX-10	HH	21.15	29.40	17.54	6.54E-03	2.44E+01	3.72E+03	2.69E-04
LX-11	HH	25.91	31.04	18.42	5.69E-02	3.59E+02	6.31E+03	1.58E-04
LX-4	HH	21.22	28.02	15.37	1.80E-02	1.15E+02	6.40E+03	1.56E-04
LX-5	HH	25.61	30.20	13.82	8.30E-02	7.05E+03	8.50E+04	1.18E-05
LX-6	HH	21.04	27.44	13.45	2.37E-02	3.85E+02	1.62E+04	6.16E-05
LX-7	HH	21.61	29.77	15.40	6.99E-03	1.47E+02	2.11E+04	4.74E-05
LX-8	HH	21.70	28.33	15.59	2.03E-02	1.39E+02	6.85E+03	1.46E-04
LX-9	HH	27.79	30.33	16.53	3.45E-01	4.92E+03	1.43E+04	7.01E-05
ML-11	HH	29.14	18.32	28.88	3.61E+03	2.39E+00	6.63E-04	1.51E+03
ML-2	HH	27.22	17.40	26.17	1.82E+03	4.14E+00	2.28E-03	4.39E+02
ML-3	HH	30.01	20.03	28.39	2.01E+03	6.14E+00	3.05E-03	3.28E+02
ML-4	HH	26.44	16.32	27.62	2.23E+03	8.85E-01	3.96E-04	2.53E+03
ML-5	HH	23.25	30.73	19.21	1.12E-02	3.30E+01	2.95E+03	3.39E-04
ML-6	HH	29.03	30.89	19.58	5.51E-01	1.40E+03	2.55E+03	3.92E-04
ML-7	HH	27.55	17.52	25.33	2.09E+03	9.30E+00	4.46E-03	2.24E+02
JN1-13 *	KM	31.73±0.23	16.46±0.03	18.16±0.04	(1.02±0.16)×10-4	3.90±0.14	3.82E+04	2.62E-05
LQ-3	KM	22.71	30.56	17.20	8.69E-03	9.14E+01	1.05E+04	9.51E-05
LQ-4	KM	28.26	35.05	20.12	1.81E-02	5.64E+02	3.12E+04	3.21E-05
LQ-5	KM	27.31	34.31	28.34	1.57E-02	9.79E-01	6.24E+01	1.60E-02
LQ-6	KM	28.79	31.65	18.23	2.76E-01	3.02E+03	1.09E+04	9.15E-05
LQ-7	KM	27.97	37.16	27.96	3.45E-03	2.01E+00	5.84E+02	1.71E-03
LQ-8	KM	28.32	18.19	28.20	2.24E+03	2.18E+00	9.75E-04	1.03E+03
SL-1	KM	28.60	36.18	25.75	1.04E-02	1.44E+01	1.38E+03	7.25E-04
SL-10	KM	29.47	32.01	18.50	3.45E-01	4.01E+03	1.16E+04	8.60E-05
SL-11	KM	29.31	32.06	18.13	2.98E-01	4.65E+03	1.56E+04	6.41E-05
SL-2	KM	21.63	31.69	17.87	1.87E-03	2.71E+01	1.45E+04	6.92E-05

SL-3	KM	26.28	30.21	15.23	1.31E-01	4.24E+03	3.23E+04	3.10E-05
SL3-13 *	KM	32.55±0.17	14.25±0.28	17.80±0.03	(4.49±0.53)×10-5	14.32±2.75	3.19E+05	3.14E-06
SL-5	KM	28.28	32.03	18.42	1.49E-01	1.86E+03	1.25E+04	8.02E-05
SL-6	KM	26.36	29.71	16.19	1.97E-01	2.31E+03	1.17E+04	8.52E-05
SL-8	KM	25.58	27.51	13.91	5.27E-01	6.52E+03	1.24E+04	8.09E-05
SL-9	KM	28.41	31.11	17.23	3.08E-01	4.65E+03	1.51E+04	6.63E-05
YL-1	KM	25.12	15.71	39.03	1.36E+03	1.30E-04	9.53E-08	1.05E+07
YL-10	KM	26.72	29.19	15.91	3.59E-01	3.58E+03	9.97E+03	1.00E-04
YL-11	KM	27.13	17.23	26.14	1.92E+03	3.98E+00	2.07E-03	4.83E+02
YL-12	KM	25.27	22.19	14.79	1.70E+01	2.86E+03	1.68E+02	5.96E-03
YL-13	KM	24.60	26.57	14.12	5.11E-01	2.84E+03	5.57E+03	1.80E-04
YL-14	KM	22.05	30.70	15.35	4.96E-03	2.08E+02	4.19E+04	2.39E-05
YL-15	KM	27.92	26.05	27.52	7.30E+00	2.63E+00	3.61E-01	2.77E+00
YL-3	KM	25.58	26.12	14.47	1.38E+00	4.43E+03	3.21E+03	3.12E-04
YL-4	KM	25.04	13.91	22.33	4.49E+03	1.31E+01	2.91E-03	3.43E+02
YL-5	KM	27.12	18.88	28.87	6.05E+02	5.96E-01	9.84E-04	1.02E+03
YL-6	KM	26.31	27.63	15.60	8.00E-01	3.35E+03	4.18E+03	2.39E-04
YL-7	KM	30.87	31.96	24.43	9.38E-01	1.74E+02	1.85E+02	5.40E-03
YL-9	KM	24.09	14.27	27.91	1.81E+03	1.41E-01	7.82E-05	1.28E+04
LC14-1	LC	23.05	26.46	13.69	1.89E-01	1.32E+03	7.00E+03	1.43E-04
LC14-10	LC	23.02	26.77	14.14	1.49E-01	9.45E+02	6.33E+03	1.58E-04
LC14-11	LC	23.17	26.60	13.34	1.86E-01	1.83E+03	9.82E+03	1.02E-04
LC14-13	LC	23.11	26.26	12.17	2.26E-01	3.93E+03	1.74E+04	5.75E-05
LC14-14	LC	23.98	26.08	13.90	4.65E-01	2.17E+03	4.67E+03	2.14E-04
LC14-15	LC	23.88	28.28	13.01	9.48E-02	3.74E+03	3.94E+04	2.54E-05
LC14-2	LC	24.48	16.14	28.24	6.47E+02	1.48E-01	2.29E-04	4.38E+03
LC14-3	LC	26.05	20.64	31.92	8.50E+01	3.40E-02	4.00E-04	2.50E+03
LC14-4	LC	24.21	27.68	14.18	1.81E-01	2.10E+03	1.16E+04	8.62E-05
LC14-5	LC	24.44	29.46	17.06	6.17E-02	3.34E+02	5.41E+03	1.85E-04
LC14-6	LC	27.12	29.63	37.98	3.50E-01	1.07E-03	3.07E-03	3.26E+02

LC14-7	LC	26.55	29.63	16.18	2.36E-01	2.64E+03	1.12E+04	8.93E-05
LC14-8	LC	27.48	34.77	20.33	1.28E-02	2.84E+02	2.22E+04	4.50E-05
LC14-9	LC	26.19	19.41	24.15	2.20E+02	8.25E+00	3.75E-02	2.67E+01
LL-1	QJ	27.00	18.02	22.56	1.01E+03	4.33E+01	4.27E-02	2.34E+01
LL-10	QJ	29.42	19.49	27.29	1.94E+03	8.75E+00	4.50E-03	2.22E+02
LL-11	QJ	30.12	27.99	16.16	8.75E+00	3.19E+04	3.64E+03	2.75E-04
LL-12	QJ	28.15	19.43	31.35	8.41E+02	2.18E-01	2.59E-04	3.87E+03
LL-2	QJ	27.92	26.09	16.27	7.12E+00	6.42E+03	9.02E+02	1.11E-03
LL-3	QJ	29.76	30.15	19.20	1.52E+00	3.01E+03	1.98E+03	5.05E-04
LL-4	QJ	29.64	30.13	18.52	1.43E+00	4.46E+03	3.13E+03	3.20E-04
LL-5	QJ	28.83	31.45	18.10	3.26E-01	3.40E+03	1.04E+04	9.59E-05
LL-6	QJ	28.54	32.31	17.86	1.47E-01	3.29E+03	2.23E+04	4.48E-05
LL-7	QJ	29.16	18.40	28.59	3.46E+03	2.98E+00	8.60E-04	1.16E+03
LL-8	QJ	28.57	18.13	25.70	2.78E+03	1.46E+01	5.25E-03	1.91E+02
LL-9	QJ	29.13	29.66	16.35	1.39E+00	1.41E+04	1.02E+04	9.85E-05
SZ-1	QJ	19.08	25.09	15.89	3.10E-02	1.83E+01	5.89E+02	1.70E-03
SZ-11	QJ	20.73	28.12	13.75	1.19E-02	2.52E+02	2.11E+04	4.73E-05
SZ-12	QJ	20.77	27.29	14.56	2.18E-02	1.48E+02	6.80E+03	1.47E-04
SZ-13	QJ	20.64	29.56	13.10	4.11E-03	3.70E+02	9.01E+04	1.11E-05
SZ-14	QJ	21.64	29.16	14.71	1.09E-02	2.44E+02	2.24E+04	4.46E-05
SZ-15	QJ	21.68	32.64	18.03	1.00E-03	2.50E+01	2.50E+04	4.00E-05
SZ-16	QJ	22.72	29.41	15.44	1.93E-02	3.10E+02	1.61E+04	6.23E-05
SZ-2	QJ	20.57	24.17	13.05	1.65E-01	3.68E+02	2.23E+03	4.48E-04
SZ-3	QJ	21.18	27.29	14.46	2.90E-02	2.11E+02	7.26E+03	1.38E-04
SZ-4	QJ	22.47	33.13	23.65	1.23E-03	8.82E-01	7.15E+02	1.40E-03
SZ-5	QJ	23.03	29.28	16.64	2.64E-02	1.67E+02	6.34E+03	1.58E-04
SZ-7	QJ	20.91	26.51	14.94	4.12E-02	1.25E+02	3.04E+03	3.29E-04
SZ-8	QJ	22.81	29.18	16.78	2.41E-02	1.31E+02	5.43E+03	1.84E-04
LT-1	XW	23.66	27.79	19.78	1.14E-01	2.94E+01	2.58E+02	3.88E-03
LT-10	XW	28.06	35.92	19.12	8.61E-03	9.80E+02	1.14E+05	8.79E-06

LT-3	XW	22.93	29.26	13.97	2.48E-02	9.92E+02	4.00E+04	2.50E-05
LT-5	XW	23.53	26.77	12.37	2.11E-01	4.57E+03	2.16E+04	4.63E-05
LT-6	XW	25.89	28.89	13.93	2.50E-01	7.96E+03	3.19E+04	3.14E-05
LT-7	XW	25.57	32.06	18.96	2.21E-02	1.95E+02	8.83E+03	1.13E-04
LT-9	XW	21.01	25.14	12.02	1.14E-01	1.01E+03	8.87E+03	1.13E-04
RS-1	XW	26.12	29.94	19.60	1.42E-01	1.83E+02	1.29E+03	7.76E-04
RS-10	XW	26.46	33.31	20.09	1.72E-02	1.64E+02	9.54E+03	1.05E-04
RS-11	XW	28.90	30.17	16.37	8.28E-01	1.18E+04	1.43E+04	6.98E-05
RS-2	XW	22.11	34.42	16.05	3.93E-04	1.33E+02	3.38E+05	2.96E-06
RS-4	XW	28.36	31.21	16.95	2.78E-01	5.43E+03	1.95E+04	5.12E-05
RS-6	XW	22.67	28.64	16.66	3.18E-02	1.29E+02	4.06E+03	2.46E-04
RS-7	XW	27.85	30.76	23.51	2.67E-01	4.07E+01	1.53E+02	6.55E-03
RS-8	XW	24.06	31.18	17.16	1.44E-02	2.40E+02	1.66E+04	6.02E-05
RS-9	XW	22.18	28.46	14.15	2.56E-02	5.20E+02	2.03E+04	4.92E-05
TT-2	XW	26.12	24.09	15.47	8.15E+00	3.20E+03	3.93E+02	2.54E-03
TT-3	XW	26.90	29.86	15.56	2.56E-01	5.16E+03	2.02E+04	4.95E-05
TT-4	XW	25.59	29.57	14.53	1.27E-01	4.28E+03	3.38E+04	2.96E-05
TT-5	XW	21.53	26.91	13.13	4.82E-02	6.75E+02	1.40E+04	7.14E-05
TT-6	XW	26.70	28.52	15.10	5.68E-01	6.20E+03	1.09E+04	9.17E-05
YM1-10	YX	25.47	31.52	15.01	3.01E-02	2.80E+03	9.33E+04	1.07E-05
YM1-12	YX	26.73	20.32	30.86	1.69E+02	1.14E-01	6.71E-04	1.49E+03
YM1-13	YX	24.94	28.64	15.57	1.54E-01	1.32E+03	8.58E+03	1.17E-04
YM1-14	YX	24.35	29.42	15.42	5.94E-02	9.77E+02	1.64E+04	6.09E-05
YM1-15	YX	24.72	16.75	25.01	5.00E+02	1.64E+00	3.28E-03	3.05E+02
YM1-17	YX	25.79	32.72	18.23	1.64E-02	3.78E+02	2.31E+04	4.34E-05
YM1-2	YX	22.11	31.79	20.42	2.45E-03	6.47E+00	2.64E+03	3.78E-04
YM1-21	YX	26.26	30.12	16.15	1.38E-01	2.22E+03	1.61E+04	6.23E-05
YM1-22	YX	25.61	22.11	34.22	2.26E+01	5.11E-03	2.26E-04	4.43E+03
YM1-24	YX	25.51	33.43	20.62	8.24E-03	5.95E+01	7.22E+03	1.39E-04
YM1-25	YX	26.00	17.10	30.06	9.55E+02	1.20E-01	1.25E-04	7.98E+03

YM1-26	YX	27.23	30.25	18.65	2.47E-01	7.64E+02	3.09E+03	3.23E-04
YM1-27	YX	26.02	19.84	32.34	1.45E+02	2.52E-02	1.73E-04	5.78E+03
YM1-28	YX	25.56	18.32	26.25	3.02E+02	1.24E+00	4.10E-03	2.44E+02
YM1-29	YX	27.42	31.89	18.32	9.03E-02	1.10E+03	1.21E+04	8.25E-05
YM1-30	YX	28.05	35.66	22.80	1.03E-02	7.62E+01	7.42E+03	1.35E-04
YM1-31	YX	29.55	36.34	25.77	1.81E-02	2.75E+01	1.52E+03	6.58E-04
YM1-32	YX	22.12	22.94	38.77	1.13E+00	1.95E-05	1.72E-05	5.81E+04
YM1-8	YX	26.65	29.70	15.45	2.42E-01	4.73E+03	1.95E+04	5.12E-05
GB192 *		17.43±0.22	24.21±0.4	17.98±0.19	1.83±0.37	(1.68±0.50)×10-2	9.18E-03	1.09E+02

* Samples from study by Wang et al. [25]
