# Supplementary figures and images for: Genetic Differentiation and Widespread Mitochondrial Heteroplasmy among Geographic Populations of the Gourmet Mushroom Thelephora ganbajun from Yunnan, China
Source: Genes (Basel). 2022 May 11;13(5):854. doi: 10.3390/genes13050854 (PMC9141859; doi:10.3390/genes13050854)

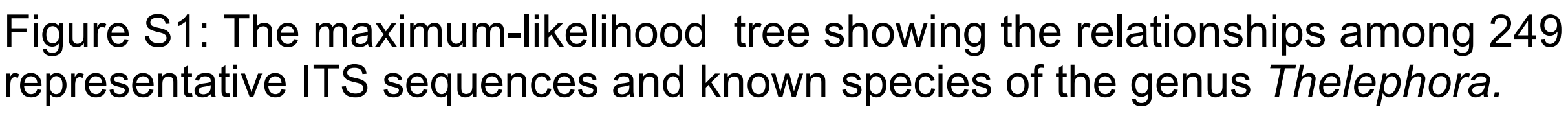

Supplement: Supplementary file 1 [file genes-13-00854-s001.zip › Figure S1 The maximum-likelihood tree-1-1.pdf]
